# Integrating Embodied Cognition and Information Processing: A Combined Model of the Role of Gesture in Children's Mathematical Environments

**DOI:** 10.3389/fpsyg.2021.650286

**Published:** 2021-04-09

**Authors:** Raychel Gordon, Geetha B. Ramani

**Affiliations:** Department of Human Development and Quantitative Methodology, University of Maryland, College Park, MD, United States

**Keywords:** gesture, math, executive function, children, learning

## Abstract

Children learn and use various strategies to solve math problems. One way children's math learning can be supported is through their use of and exposure to hand gestures. Children's self-produced gestures can reveal unique, math-relevant knowledge that is not contained in their speech. Additionally, these gestures can assist with their math learning and problem solving by supporting their cognitive processes, such as executive function. The gestures that children observe during math instructions are also linked to supporting cognition. Specifically, children are better able to learn, retain, and generalize knowledge about math when that information is presented within the gestures that accompany an instructor's speech. To date, no conceptual model provides an outline regarding how these gestures and the math environment are connected, nor how they may interact with children's underlying cognitive capacities such as their executive function. In this review, we propose a new model based on an integration of the information processing approach and theory of embodied cognition. We provide an in-depth review of the related literature and consider how prior research aligns with each link within the proposed model. Finally, we discuss the utility of the proposed model as it pertains to future research endeavors.

## Introduction

Hand gestures are used in a variety of mathematical contexts by children and adults alike. These gestures include both directed, meaningful movements intended to convey information, as well as less formal shifting of the hands that occurs naturally alongside conversation. Children use gestures to represent information, enhance conversation, and even support their own cognition (for a review, see Goldin-Meadow, 2009). Children's self-produced gestures (e.g., Broaders et al., 2007; Cook et al., 2008) as well as the gestures they see teachers use during instruction (e.g., Singer and Goldin-Meadow, 2005) have been shown to support their math learning. Given that children's early math knowledge has been consistently linked with their later math achievement (Claesens and Engel, 2013; Geary et al., 2013; Watts et al., 2014; Geary and Vanmarle, 2016), factors related to children's math understanding, like gesture, are important to understand.

One way that gestures can support mathematical learning is through their ability to aid children's cognition (Goldin-Meadow et al., 2001; Cook et al., 2012). Specifically, gesture can be linked to different components of domain-general abilities, such as executive function (EF). EF refers to the cognitive control processes that coordinate sub-processes such as attention shifting, working memory, and inhibitory control (e.g., Bull and Lee, 2014). Given the association between children's EF and math abilities (see Clements et al., 2016 for a review), as well as gestures supporting math through EF, we propose that it is important to study these factors together.

In this review, we discuss how children's use of and exposure to hand gestures during math contexts can shape their learning. Within the first section, we provide background information on two prevalent, but separate theories; information processing approach and theory of embodied cognition. We assert that combining these theories allows for a better understanding of the dynamic relations of gesture, EF, and children's mathematical learning. Thus, we propose a new model based on a combination of central tenets from these two theories. Next, we review relevant literature, and discuss how these results may be interpreted within the proposed model. In the final sections, we present opportunities for future empirical work. This paper serves as a unique examination of prior research through the lens of a unified model.

### Overview of Gestures and Gesture Theories

Gestures are dynamic hand and body movements which accompany language. They can occur spontaneously or intentionally, and oftentimes provide different yet complementary information to a person's speech (Church and Goldin-Meadow, 1986; McNeill, 1992; Church, 1999). A speaker's gestures can facilitate listener's comprehension (for a meta-analysis, see Hostetter, 2011) and improve overall communication compared to speech alone (Church et al., 2000). Therefore, information provided by speakers' gestures are useful to those who see them.

Self-produced gestures can serve an important, internal purpose for the user as well. Hand gestures allow a speaker to simultaneously process their thoughts and put them into communicative form (McNeill, 1992; Krauss, 1998). People continue to gesture even when no one is watching (Krauss et al., 1995; Alibali et al., 2001). Research has shown that congenitally blind speakers use gestures even when they are communicating with a blind listener (Iverson and Goldin-Meadow, 1998), suggesting even those who have never seen gestures modeled in communication will use them too. Thus, gestures appear to support internal mechanisms of communication and cognition.

Due to the prevalence of gestures across ages, contexts, and domains, numerous theoretical models have been created to account for their communicative and cognitive functions. Each theory understandably overlaps in part with another; however, each one also provides complementary information explaining new contexts, factors, and functions. For example, frameworks that focus on what we can uncover about the speaker (Goldin-Meadow, 2003), or where these gestures emerge from (Gesture as Simulated Action framework, GSA; Hostetter and Alibali, 2008) both provide insight into how gestures relate to and shape underlying cognitive processes. Furthermore, the GSA framework builds upon another foundational idea that these cognitive processes are rooted within the environment (Embodied cognition, Barsalou, 1999). Gestures have also previously been considered under theories of cognition, such as Cognitive Load Theory (Sweller, 1988). This framework provides an explanation that self-produced gestures reduce “cognitive load,” a mechanism that is often considered as one of the main roles of gestures. Each of these, as well as other gesture-related frameworks, provide unique and compelling explanations of the distinctive roles of gestures as they relate to a particular set of circumstances. However, these models do not consider the specific role of gestures in mathematical contexts. Our goal was to create a model based on the growing literature regarding the benefits of gestures, both produced and seen by children, during math environments.

### A New Model of Gesture for Math Learning

We propose that the literature is best supported by a model that integrates two previously established frameworks. First is the information processing approach, commonly used within math research to represent how information moves through each component of human cognition during problem solving and learning. Second is the theory of embodied cognition, the basis of many gesture theories. This framework provides our model's infrastructure, as it articulates the importance of human-cognition being situated within a body, further encompassed in an active, stimuli-ridden environment.

#### Information Processing Approach

One way to conceptualize how children solve math problems and learn math related content is the Information Processing Approach (IP; e.g. Pellegrino and Goldman, 1987). This is not a single theory, rather an umbrella term for approaches which explains human cognition as a system that processes stimuli input from the environment and delivers a variety of outputs. The IP model suggests that learning occurs via a flow of information through a series of memory stores and processes. These distinctive elements in the IP approach can be conceptualized as the subcomponents of EF (adapted from Lutz and Huitt, 2003). Input is received from stimuli in the environment by way of the sensory registry. Attention is directed to fixate on relevant information, which progresses to working memory, a short-term store where information is held and processed for use in further cognitive tasks (Gathercole, 1998). Working memory is responsible for determining what information is important, choosing and enacting problem-solving strategies, and coming to a solution. Ultimately, information will be either be forgotten or encoded and stored in long-term memory for retrieval at a later time.

Although the IP framework can be broadly applied to represent children's math problem solving and learning, there are ways in which it could be further specified. First, a framework that focuses on both visual and auditory math-specific input could help to better understand how this input is relevant for children's math abilities and learning. Second, when investigating the role of gesture for children's early mathematics, it is important for a framework to include the body itself. While the IP model describes the cognitive processes, it does not explain any co-occurring physical behaviors. Thus, this framework cannot adequately account for the gesture-specific benefits that may occur within a math-related context. The question remains open as to how to model the role the learner's body, and the different types of math stimuli (words and gestures) within the environment.

#### Embodied Cognition

One theory that provides insight into these two components is embodied cognition (EC). While EC has been conceptualized in various ways, each adaptation generally emphasizes the body and stimuli within the surrounding environment as important to cognition (e.g., Barsalou, 1999; Clark, 1999; Shapiro, 2019). Here, we outline Wilson's (2002) presentation of EC. Specifically, she conveys six central claims of EC, three of which outline the importance of considering cognition as a situated process, and the other three focus on the importance of the body as a tool for cognition.

The first claim stipulates that cognition is situated. In other words, cognitive processing occurs in parallel with the task-relevant inputs and outputs from the environment. Thus, cognition cannot be separated from interplay between perception of the environment and subsequent actions taken. The second claim is that cognition is “time pressured,” where cognitive processing requires real-time responses to the stimuli in their environment. Lastly, Wilson's fourth claim states that the environment is an important part of the cognitive system. Though similar to the first claim, Wilson outlines that since the reception of stimuli, cognitive processes, and behavioral responses are cyclical in nature, each of these components cannot be considered alone.

Wilson's claims three, five, and six all focus on the role of the individual's body in cognition. Claim three emphasizes that humans tend to off-load cognitive work externally in strategic ways. Wilson provides finger counting as an example, indicating this gesture can be used as a representation of relevant numeric information (e.g., linking number words to objects to keep track of quantity). Thus, offloading is a critical cognitive function that helps the speaker reason and express thoughts. The fifth claim states that cognition's primary function is for action. Meaning, a person's perception of the world as well as their concepts and memory are both “for” and “formulated by” their behaviors. Lastly, claim six says that off-line cognition is “body-based.” Wilson's conceptualization of off-line cognitive processes involves any that are separable from the time-sensitive environment. Importantly, though they are distinct from the environment itself, the processes within the mind are inevitably tied to cognitive mechanisms that were originally designed for external behaviors, such as sensory processing and motor control.

The critical takeaway from Wilson's presentation of EC is that both the body and environment are integral to cognition. Her representation of EC underscores how embodied practices can result in an offloading of cognitive load. Based on how EC provides the important contribution of the body and the environment, and a focus on how cognition may be offloaded, we propose a model combining central tenets from both IP and EC.

#### The Proposed Model

**Figure 1:**
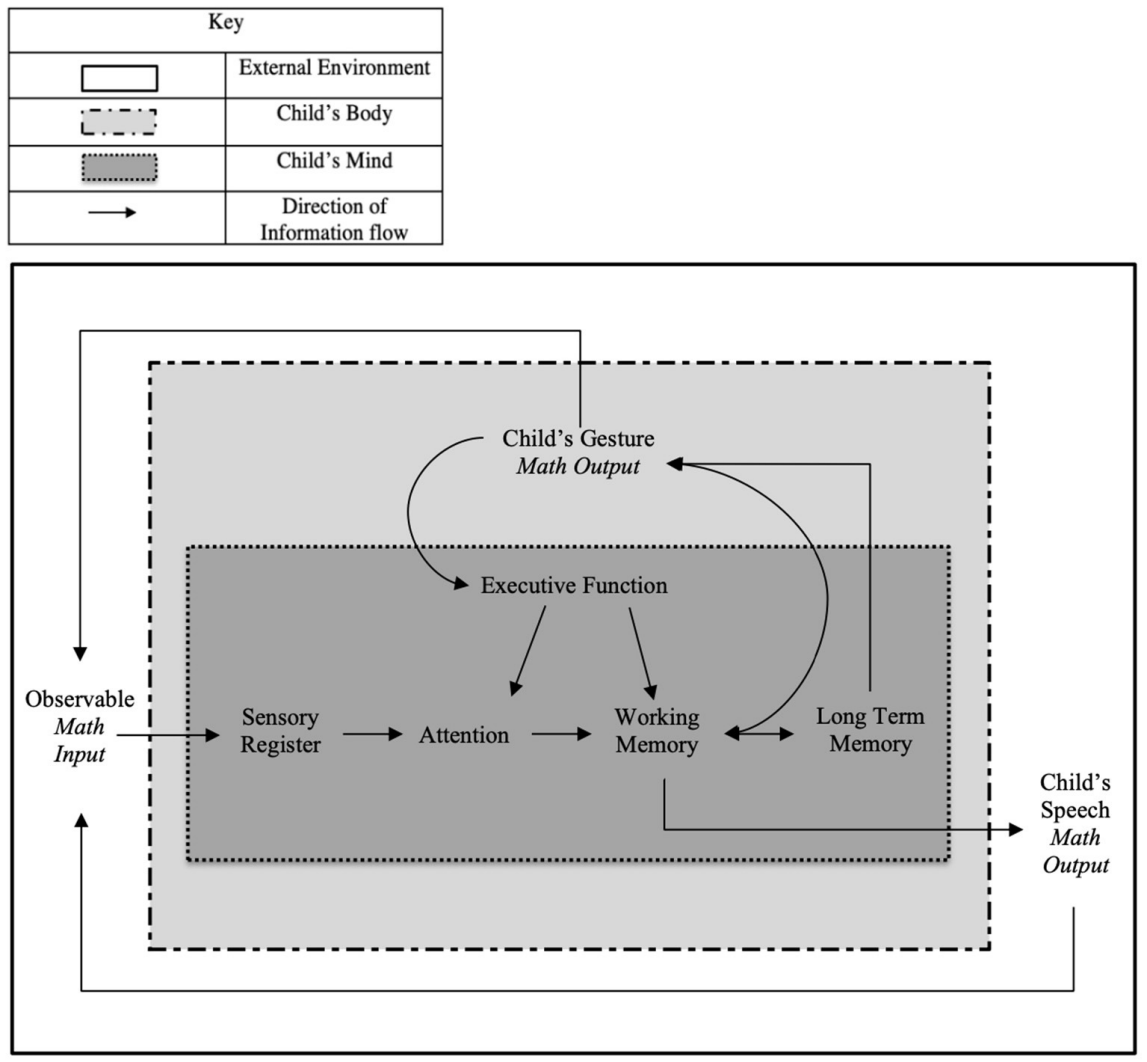
Combined model of information processing and embodied cognition.

The proposed model contains aspects from EC and IP, and specific contextual elements of gestures within the mathematical environment (Figure 1).

The proposed model is unique in its applicability to different math domains. For example, during a lesson on addition, math input could include a teacher's speech and gestures in reference to an equation on the chalkboard, while the output could be children's verbal and gestural response and explanations. In another context where a younger child is counting a set of objects, their math-input could be instructions and countable objects, and their output may include them pointing and counting out loud. Thus, there are numerous opportunities for applying the model for research by specifying the components (learner, input, and output) within a math environment.

Notably, our model also does not differentiate between perceived speech and gestures. Instead, it includes a unified representation of math-input. We base this combined representation on research showing that simultaneous presentation of these two observed modalities can be beneficial for children (Congdon et al., 2017). However, children's math-output is differentiated in the model because the literature (reviewed in subsequent sections) suggests children's gesture and speech often contain different but complementary information. For example, recent work supports separation of math output by modality, given that temporal-synchrony of self-produced gestures and speech does not relate to learning and retention for children in the same way that observed gestures do (Wakefield et al., 2021).

Incorporating gesture as input and output separately allows the model to be adapted in two critical ways. First, it can be applied to different mathematical domains (e.g., cardinality, algebra, fractions, etc.), such that the input and output can vary by content. Second, the model can be used to understand a broad range of differences in children's general EF abilities and math knowledge specifically. This is of particular importance given children are found to be adaptive in their responses to math problems (Siegler et al., 1996), and that the strategies children display may differ between their speech and gesture (Goldin-Meadow et al., 1993b).

Consider the example of a child solving the problem 3+2; if they have the answer memorized, they may quickly answer “5!” using a direct retrieval strategy of relevant math knowledge. A second child, who has only learned about addition principles generally, would likely respond differently to the same problem. They may use a backup strategy (i.e., any method other than retrieval), such as holding up three fingers then extending two more while counting on “4…5! The answer is 5!.” The proposed model highlights how these children's individual differences in math knowledge could impact their use of self-produced gestures, and would allow researchers to explore the theoretical implications of how these strategies connect to their subsequent math abilities and later learning.

In addition to understanding the connection to math knowledge, an additional goal of the proposed model is to explain how gestures may be beneficial for EF and its subcomponents. EF includes three separate, but interrelated processes; attention shifting, inhibitory control, and working memory (Miyake et al., 2000). While EF is often discussed as a multidimensional construct, there is also evidence of unidimensionality in early childhood (Wiebe et al., 2008; Hughes et al., 2010). This makes it difficult to determine empirically whether the benefits of gesture for children relate to EF broadly, or to one specific sub-component. For example, it is common within the gesture literature to discuss gestures as providing a reduction in “cognitive load” (Goldin-Meadow et al., 2001) or linking them to executive function (EF) broadly (O'Neill and Miller, 2013). As such, connections between sub-components of EF and gesture are represented in the current review based on how they are discussed within their respective studies. The implications of this approach are reviewed in the discussion.

In sum, when studying the role of gesture in math environments, we propose a combined model of the IP approach and theory of EC. By establishing the pattern of information flow from the IP model within specific embodied locales and conventions of EC, this new model provides a dynamic representation of the cognitive impact of gestures in a mathematical environment. The connections between children's domain-specific knowledge (stored in long-term memory) to their self-produced gestures are illustrated within the model itself, as is an additional pathway between math-input and children's EF. Thus, both types of gestures are connected with children's cognition. Each of these connections will be considered through a review of the current literature.

### Goals of the Current Review

There are two primary goals of the current review. First, we will review empirical work on the relations between children's mathematics ability and EF; mathematics ability and gesture use; as well as their gesture use and EF. Each of these will be discussed in terms of how this research fits within the proposed model. The second goal is to address any remaining gaps within the literature in order to demonstrate how the proposed model lays the foundation for future research to examine the mechanistic role that gesture may play in children's math learning.

### Search Methodology

The present review is focused on connecting three separate, but related, bodies of literature: the gestures which children see and use, their EF abilities, and their mathematics knowledge. In the present review, less time is spent on connections between children's mathematics and EF abilities, given the numerous reviews of this topic that are readily available (for reviews, see Bull and Espy, 2006; Bull and Lee, 2014; and Cragg and Gilmore, 2014). Each of the subsequent components (gesture and math, gesture and EF) were investigated in a review conducted with APA PsycInfo database and Google Scholar in February through March of 2020. Follow-up searches were conducted in June-July of 2020. Relevant articles that came to our attention after our two initial searches were also included. In order to be included, studies must have been (1) published in English; (2) reports of original research, conceptualization of theory, or related reviews of literature published in peer-reviewed journals; (3) focused primarily on outcomes with children. Each search was conducted with separate keyword searches. Math and executive function were searched along with keyword combinations including but not limited to children, learning, and review. Math and gesture were searched along with keyword combinations including but not limited to children, learning, education, instruction, teacher, and review. Lastly, gesture and executive function were searched along with keyword combinations including but not limited to children, individual differences, working memory, attention, inhibition, childhood, and review.

## Math and Executive Function

Numerous studies have demonstrated a relation between children's mathematics ability and their EF (for reviews, see Bull and Espy, 2006; Bull and Lee, 2014; Cragg and Gilmore, 2014; Jacob and Parkinson, 2015; Peng et al., 2016). Broadly, this relation is consistent across different mathematical areas, including early numerical tasks, arithmetic problems, word problems, and standardized math measures (e.g., Lee et al., 2009, Bull et al., 2011; Van der Ven et al., 2012). It is critical to note that in both empirical and applied settings, EF has been conceptualized in numerous ways with researchers using a variety of assessment measures. As a result, empirical work on relations between math and EF are extensive and this literature has been previously reviewed as noted. Therefore, the focus of this section is to briefly summarize this research to demonstrate how representation of EF within the proposed model provides a specific operational system that is firmly connected to math contexts throughout childhood.

Cross-sectional correlational research has shown that different sub-components of EF are related to children's mathematical performance. For example, research indicates that working memory abilities are related to a range of mathematical tasks, such as early numeracy abilities (Kroesbergen et al., 2009), arithmetic achievement (Navarro et al., 2011), problem solving more broadly (Swanson, 2004), written and verbal calculation (Andersson, 2008), as well as mathematical word problem accuracy (Andersson, 2007; Zheng et al., 2011). Similar findings have shown connections between children's inhibitory control abilities and their math performance and achievement (Espy et al., 2004; Brock et al., 2009; Gilmore et al., 2013). There is additional evidence that inhibitory control, attention shifting, and working memory independently account for separate variance in children's math ability (Bull and Scerif, 2001). Further, when different sub-components of EF were examined in parallel, unique contributions of each on children's math ability were still prevalent (e.g., Bull and Scerif, 2001; St Clair-Thompson and Gathercole, 2006; Kroesbergen et al., 2009). Thus, evidence demonstrates relations between all three sub-components of EF and mathematics in children, lending support to including these factors within our model.

As children's mathematical knowledge develops, the impact of EF ability on their learning and performance differs. For children, it appears that working memory is of particular importance. Specifically, both children's symbolic (Caviola et al., 2012) and non-symbolic math abilities (Xenidou-Dervou et al., 2013) are positively related to their working memory. Importantly, children appear to rely more on their working memory than adults while solving math problems (Cragg et al., 2017). This may be due in part to how children's enactment of strategies is a more active and less efficient process and so their ability to enact a problem-solving strategy may be more of a direct result of their EF abilities compared to adults. Further, different EF abilities may allow an individual to enact different mathematical strategies (Imbo and Vandierendonck, 2007). For example, first grade children with higher working memory abilities were found to use more correct and sophisticated strategies on arithmetic problems compared to children with lower working memory capacity (Geary et al., 2012). These findings suggest that the relevance, contribution, and demand of working memory and broader EF abilities may shift depending on both the mathematical content and children's task knowledge, which can impact overall task performance. Thus, individual variation in EF abilities is a critical component to include in a model of children's math performance and learning, which is reflected in the centralized location of the proposed model.

Lastly, longitudinal studies have shown that children's EF is not only predictive of later mathematics performance (Alloway and Alloway, 2010; Monette et al., 2011), but also of their growth in mathematical abilities (Geary, 2011; Clark et al., 2013; LeFevre et al., 2013). For example, in a study following children from kindergarten to third grade, working memory related to children's early and later number competencies, which contributed to their math achievement (Krajewski and Schneider, 2009). However, training studies have shown mixed results. Some studies have found that EF training can improve children's numerical knowledge (Holmes et al., 2009; St Clair-Thompson et al., 2010; Holmes and Gathercole, 2014; Ramani et al., 2017, 2019). For example, training WM improved kindergarten children's counting skills, and WM games that included both numerical and non-numerical information improved children's counting and numerical comparison skills (Kroesbergen et al., 2014). However, others have found that providing children with EF training does not necessarily improve their mathematical knowledge (Jaeggi et al., 2012; Shipstead et al., 2012; Karbach et al., 2015). These findings suggest varying levels of efficacy in EF training on improvements in mathematics and provide the first window of opportunity for future research using the proposed model.

Overall, there is consistent cross-sectional and longitudinal evidence of relations between EF and mathematical achievement in children. These connections are found in a variety of mathematics domains, and the individual differences in EF abilities can influence children's mathematical performance. However, experimental evidence demonstrating that training EF can improve children's mathematical knowledge is less consistent, although numerous studies have shown promising results.

## Gesture and Math

In this section, we review literature on two types of gestures included in our model. First, we outline literature pertaining to gestures used by other people, such as a teacher or experimenter, to explain or teach math concepts; included in the model's “Math Input” section. Second, literature regarding children's self-produced gestures is reviewed; included in the model's “Math Output” section. Studies in these areas establish two critical functions (represented by connected arrows in Figure 1). One function highlights how children's self-produced gestures may convey math information (stored within their memory), which assists with their cognitive processing (EF). A second connection between children's gesture math-output connects back to their math input, which allows for the possibility that children's gestures elicit math information from their environment. Each of these are functions are reviewed and discussed.

### Math Input: Observing Other People's Gestures

Individuals who observe a speaker's gestures during a mathematical context can extract useful information (Goldin-Meadow et al., 1992; Alibali et al., 1997; Kelly and Church, 1998). No training is required to gather this information, as children are readily able to attend to information found uniquely in gesture (Kelly and Church, 1997). Therefore, gestures that occur within math environments are straightforward in their presentation yet are critical to understand.

Experimental studies have shown that watching gestures can support learning and generalization of math concepts. For example, Graham (1999) had 2–4-year-old children (*n* = 85) watch a puppet point while counting objects. When asked about the puppet's performance, children spoke about the puppet's gestures suggesting that from a young age, children are explicitly able to recognize gesture strategies (pointing) in a math environment. Alibali and DiRusso (1999) used a similar paradigm with preschoolers (*n* = 20; M age = 4.67), where a subset of children was asked to count aloud while watching a puppet gesture to keep track of the objects. These children made fewer counting errors compared to children who had no gesture supports (either their own, or the puppets). These studies illustrate how young children can benefit from receiving gestures as part of their math input.

Research has also examined how gesture input could benefit other domains of math. Valenzeno et al. (2003) worked with 25 preschool-age children (M age = 4.5 years) who watched videos of teachers providing instruction on symmetry in a speech alone, or in gesture plus speech. Children who saw the gesture plus speech instructions had higher posttest scores for this math-concept, compared to children who received instruction in speech alone. Thus, children who received math-input with gesture showed greater improvement in math knowledge compared to their peers who received speech alone.

Additionally, children are also able to detect information that is uniquely communicated through gestural math-input. Specifically, Goldin-Meadow et al. (1999) asked a group of teachers to give children (*n* = 49, M age = 9.83 years) lessons on mathematical equivalence^1^. Teachers were not specifically told to gesture, though they did gesture spontaneously during instruction. These gestures contained relevant problem-solving strategies, such as a v-handshape to group two numbers together visually that should be summed, or gesticulating a flat palm under one side of a problem and then the other to indicate equality. These gestures either reinforced the information in the teacher's speech (gesture-speech match) or contained different, but complementary information (gesture-speech mismatch). Overall, children were more likely to reiterate their teacher's speech if it was accompanied by a gesture. Critically, children were also found to be able to recognize information that was solely presented within a teacher's gesture. This suggests that children both notice and process the mathematical information presented uniquely by gestures.

Children's ability to perceive information from gesture is further supported by evidence from a bilingual sample (Church et al., 2004). In this study, 51 Spanish-speaking first grade students (M age = 7.0 years) were assigned either to a Spanish-speaking classroom in school, or to an English-speaking classroom. Students watched a video of an English-speaking teacher providing instructions either with or without gestures. These gestures were gesture-speech mismatches, such that they contained unique but complementary information to speech. Overall, children in both classrooms benefited from the inclusion of gesture during instruction, and Spanish-speaking children's learning in particular increased from 0 to 50%. This suggests an additional benefit of including gestures as math-input. Specifically, gestures may be a more universally accessible representation of math information, as its manual format is not tied to a language.

Singer and Goldin-Meadow (2005) continued to build off this line of inquiry using the mathematical equivalence paradigm. Specifically, 3rd and 4th grade children (*n* = 160) were taught problem-solving strategies either with no gesture, gesture-speech matches, or gesture-speech mismatches. Children were more likely to learn when their teacher's math-input contained one problem-solving strategy in speech, while simultaneously presenting a different strategy in gesture. This finding extends previous work by suggesting that the addition of gesture to speech is unique in its ability to present two math concepts simultaneously (one in each modality), which in turn facilitates learning. Therefore, the inclusion of gestures as an accessible, beneficial form of math-input is cemented in the model.

It is additionally important to review research on how gesture input may impact children's math knowledge. Cook et al. (2013) asked 7–10-year-old children (*n* = 184) to watch a video where an instructor provided a lesson on math equivalence either with or without gestures. Children completed both an immediate and delayed posttest to test for general learning and transfer. Compared to children who received instruction in speech alone, children who received gestural math-input performed better on both the immediate and later posttests, including a transfer of knowledge to new problems. Thus, children appear to gain knowledge quicker and to generalize knowledge better when that information is provided with supporting gestures, as opposed to speech alone. These findings provide insight into how the inclusion of gestural math-input could impact children's own math-output, such as their response to a later math test.

Additional work expanded on these results with a computerized avatar (Cook et al., 2017). Sixty-five children (M age = 9.0) watched as a computer avatar provided instruction on mathematical equivalence, either with or without accompanying gestures. Children who saw the gesturing avatar learned more, solved problems quicker, and were more likely to generalize their knowledge. Thus, children benefited from the addition of gesture regardless of whether their instructor was human or a computer avatar. These results reveal how gestural math-input can be expanded to include technology-based instruction to advance children's learning and generalization of knowledge. This emphasizes the connections within the proposed model regarding math-input to children's overall math understanding.

Together, these findings suggest that children notice, and process mathematical information provided in instructor's gesture. These gestures are found to enhance children's learning and support broader understanding through concept transfer and generalization. This literature is consistent with the proposed model; children receive math input from their instructor's gestures and speech, which supports their problem solving and later learning in the form of math-output.

However, it is also critical to understand the mechanisms by which gestures provide these supports. One study assessed this issue by manipulating whether task-objects were in view, and thus referenceable, by their subjects (Ping and Goldin-Meadow, 2008). Specifically, kindergarten and first-grade students (*n* = 61, 5–7 years old) participated in Piagetian conservation tasks where they were shown two objects (e.g., two glasses with equal liquid) and were asked if they were equal. One of the objects was manipulated, (e.g., poured into a shorter glass), such that children's understanding of conservation could be assessed when asked to explain if they were equal now. Children then received instruction on conservation, either in speech alone or gesture-plus-speech, as well as either with or without the objects present. On average, children were more likely to learn from instruction that contained gesture-plus-speech, even when the objects themselves were not present. In other words, gestural math-input was helpful beyond the scope of referencing specific, concrete objects within children's environment. Thus, the function of gesture as math-input goes beyond simple attention direction or grounding of speech in the physical environment and has broader implications for children's learning.

Overall, the literature suggests that the gestures which children observe as math-input can directly support their math learning, which reinforces these connections in the proposed model. Children are better able to learn, retain, and generalize new information about math when their instructor uses both gestures and speech, compared to speech alone. When children cannot access math-information in their teacher's speech, gestures become even more important. These benefits extend beyond a simple direction of attention, as gestures continue to be beneficial even when the relevant items are not present.

### Math Output: Children's Self-Produced Gestures

In the following section, we review literature on the self-produced gestures children use in math contexts to scaffold their own knowledge and learning. These gestures occur spontaneously (e.g., Crowder and Newman, 1993) or as resulting from explicit instruction (e.g., Alibali and Goldin-Meadow, 1993). In the proposed model, children's own gestures are linked to supporting their ongoing cognitive functions, while also producing a form of math output. This output can then be observed by teachers to continue to inform the child's math environment (e.g. Gibson et al., 2019). Each of these functions of children's self-produced gestures are examined in turn.

Self-produced gestures have been shown to reduce cognitive load during math contexts. This benefit of gesture was examined by Goldin-Meadow et al. (2001), who asked participants to solve and explain age-appropriate math problem (e.g., math equivalence problems for children, harder problems for adults). They were also asked to remember a string of letters or words while providing the explanation for their solution. Gesture was manipulated directly, such that participants were given instruction regarding whether gestures were permitted, or if they should keep their hands on the table. Both adults and children were able to remember significantly more of their list when they used gestures during their math explanations. This finding supports the inclusion of children's self-produced gestures within the model. Furthermore, the authors suggest that the observed cognitive benefit may be due in part to gestures' utility in reducing memory demands, which may additionally link self-produced gestures to the memory processes in children's minds. Thus, this study is discussed briefly a second time in relation to working memory.

Another study investigated how self-produced gestures may further support children's performance on a math task. Specifically, Gordon et al. (2019) investigated how preschool children's own gestures may support their knowledge and performance on a cardinality task. Results indicated that children's cardinality knowledge was positively related to their spontaneous gesture use, even while controlling for age. This relation was not just driven by children who had mastered cardinality; indeed, the same positive relation between gesture and cardinality knowledge existed for the subsample of children who were still learning principles of cardinality. Children were also found to gesture the most during parts of the task that were most difficult for them, subjectively, based on their current cardinality knowledge. This emphasizes the connection in the model between children's own gestures, their math knowledge in long-term memory, facilitated by the problem-solving abilities within other components of EF.

Based on the advantages of self-produced gestures, additional work considers how providing explicit gesture instruction or encouragement to children to may impact their performance or learning in math environments. Broaders et al. (2007) examined this phenomenon in two studies with 3rd and 4th grade children who were asked to solve math equivalence problems. In the first study, children were asked to explain their solutions to these problems either using specifically with gesture, specifically without any gesture, or heard no mention of gesture. Children who were told to gesture conveyed different information in this modality (i.e., gesture-speech mismatch), such that their math-output contained new and relevant information. Therefore, instructing the use of gesture can lead children to express math knowledge with their hands that may not otherwise be communicated with their speech. The authors also sought to address whether children who received this instruction would be more receptive to learning by testing a new set of 3rd and 4th graders using a similar protocol for their second study. Results indicated that instructing children to use gesture not only taps into their implicit math-knowledge, but also makes them more likely to learn. Taken together, these results highlight how a combination of direct instruction (math-input) and the resulting self-produced gesture (math-output) could impact later math learning; the overall goal of the proposed model.

To further parse apart the benefits of instructed gestures, Goldin-Meadow et al. (2009) investigated whether specific types of gestures were more advantageous than others. Third and fourth graders completed math equivalence problems and were assigned to one of three training groups: no-gesture, correct-gesture, or partially correct gesture^2^. Overall, children learned more when any gesture was used, regardless of whether the information it contained was mathematically correct. However, children who received correct-gesture training solved more problems correctly compared to the partially correct gesture group. This suggests that gestures which contain specific, correct math information are superior to other gestural types. Furthermore, children were able to verbalize the grouping strategy used in gesture without any direct instruction, indicating that children learned a strategy from their own gestures. Taken together, these results indicate that while any gesture may benefit children, instructing specific gestures that align with math-concepts could allow children to extract and learn that information. This further supports the proposed model; children's self-produced gestures, while labeled as a form of “math-output,” have connections to and from the knowledge storage and EF processes within their minds. Thus, by providing instruction to children to self-produce a specific type of gesture, they may be able to tap into and build on task-relevant knowledge.

New research involving fMRI methods builds on the mounting evidence that providing instruction to children to use gesture improves their mathematics ability. Wakefield et al. (2019) worked with 7–9-year-old children who had engaged in the same mathematical equivalence training outlined in previous research (Cook et al., 2008; Goldin-Meadow et al., 2009). Children solved a series of these problems, then received training to express an equalizer strategy in either speech alone or speech plus gesture. Only children who had gotten all problems wrong initially then successfully solved at least half the problems after training were included in the final sample (*n* = 20). A week later, this sub-sample of children completed a short training refresher before participating in an fMRI session where they solved new mathematical equivalence problems. Results showed differences in neural activation during problem solving by training condition, such that children in the speech and gesture condition had greater activation of the motor regions of their brains compared to speech-alone. This indicates that training math concepts through self-produced gestures may have lasting neural impacts, even though children were unable to use gesture during the fMRI reading itself. Thus, the neurological research is consistent in its support for the pathways generated by the behavioral research for the proposed model.

However, it is essential to address whether these benefits are unique to gesture, or if any movement or action could render the same benefits. For example, could children use a bodily strategy consisting solely of actions and have the same mathematical benefits? Novack et al. (2014) explored this idea with 3rd grade children using the math equivalence paradigm. Children were taught to use either a physical action on objects, a concrete gesture which mimicked that action, or an abstract gesture while solving the problem. While each of these strategies lead to more learning, only children who used gestures were able to generalize their knowledge to successfully complete later problems. Therefore, given that it is gesture rather physical action that best assists learning and knowledge transfer, the current model provides a unique vantage point to delve further into how gesture confers these benefits.

Building off this line of work, Congdon et al. (2018) investigated how individual differences in children's math knowledge influenced their learning from gesture or action strategies. First grade children's initial measurement knowledge was assessed, after which they received one of four trainings for a measurement task. Half of the conditions used a physical stick above a ruler aligned with zero, the other half shifted over to align with a different whole number. Conditions were further split by action or gesture-based trainings; Action-based trainings with physical manipulatives to show children how the ruler segments could be used to count, and gesture-based training using a “pinching” gesture to highlight the relevant segments of the ruler. Children who used simpler strategies incorrectly during the initial measurement assessment benefited from the action training, but not the gesture training. However, children who initially used a more complex, but incorrect, strategy at pretest learned from both the training with actions and gestures. This finding highlights the importance of recognizing how and when gestures could be applied, as well as how individual differences in children's own math knowledge may influence the benefits of gesture. In particular, encouraging the use of gesture may help a child who has reached the particular level of underlying math knowledge, yet hinder another less-advanced child at the same time. Thus, our model centralizes the importance of gesture while also highlighting the importance of not separating the utility of the tool from its intended user.

In educational settings, it is also important to understand how children's self-produced gestures can provide information to an observer, and how this observer could provide additional math-relevant input. In their seminal work, Church and Goldin-Meadow (1986) examined 5–8-year-old children's speech-gesture mismatches to investigate whether these movements indexed their transitional knowledge. In the first study (*n* = 28), children participated in a series of Piagetian conservation tasks where an experimenter made visual transformations of two equivalent objects. Throughout the task, children were asked if the objects had the same amount and to provide an explanation after the transformation. Children were categorized as a conserver (e.g., recognized the key concept of conservation), partial conserver, or a non-conserver based on their explanations. Children's speech and gesture use were coded during their explanation to determine if they were a match or a mismatch^3^. On average, children who had more mismatches showed more complex knowledge in their gestures than their speech. Based on this finding, the authors conducted a second study where half of the children received direct instruction on the concept of equivalence while the other half were given the opportunity to physically manipulate the objects. Children who had more speech-gesture mismatches in their explanations were more likely to learn new information after training and benefited from the opportunity to play and manipulate the objects afterwards. In contrast, those children with more matches than mismatches did not show any additional benefits from explicit training or more informal contact with the objects.

These findings were further expanded upon by Perry et al. (1988), who sought to explore how spontaneous self-produced gestures used in math contexts could index children's “readiness” to learn new information. In a series of studies, they asked 9–12-year-old children to solve problems and explain their solutions related to concepts of mathematical equivalence and Piagetian conservation. In general, children's speech and gestures were more likely to match during the conceptually easier mathematical task (conservation), but more likely to mismatch during the more difficult mathematical task (mathematical equivalence). Additionally, the amount and the type of mismatches produced by children provided an index of their “readiness” to learn. Specifically, the authors suggest that children's math-output (gesture and speech) provides insight into their math knowledge, as well as whether they may be able to receive new math-input from their environment. Indeed, children's gesture and speech mismatches have been linked to their zone of proximal development (Goldin-Meadow et al., 1993a). In other words, their gestures may be used by adults to specifically calibrate future math-input to a child's individual level of understanding.

To further understand how children's self-produced gestures mark their conceptual knowledge, Garber et al. (1998) assessed the speech gesture mismatches produced by 4th grade children in their explanations of mathematical equivalence problems. Children subsequently were asked to judge the acceptability of a variety of other commonly used problem-solving strategies, some of which were incorrect. Overall, children gave the highest rating to strategies which contained conceptual elements that they had only indicated in their gestures during their initial explanations of how to solve equivalence problems. Thus, these children not only expressed knowledge uniquely in their gestures, but this knowledge was accessible when presented to them later as additional mathematical input. Therefore, by watching the gestures that children produce as a type of mathematical output, it is possible to map out what math concepts they may already have some knowledge of implicitly. Taken together, these studies findings are consistent with the proposed model; that the gestures which children produce as a form of math output are linked to the knowledge stored within their long-term memory.

These markers of conceptual knowledge are found for other domains of math knowledge too. Specifically, Gunderson et al. (2015) studied 3–5-year-old children's mismatches in the context of cardinality, an early math concept which involves an extended learning process. Children who were still in the process of learning about this concept were more than twice as accurate in their gesture responses compared to their speech. Moreover, the gestures children produced were more accurate when the information in their gestures was a mismatch with their speech. Therefore, even young children who are in the process of learning a basic numerical concept provide unique information in their gestures that is not otherwise found in their speech. This finding supports that the current model may be extended to consider mathematics more broadly, as the patterns and information in gestural mismatches appear in the form of gestural math-output with younger children as well.

There is also evidence of this phenomenon in manual languages, such as American Sign Language (ASL). Goldin-Meadow et al. (2012) examined how the gestures produced by ASL-signing deaf children (*n* = 40) in the previously explained mathematical equivalence paradigm predicted whether they would benefit from explicit instruction on those problems. In general, the children who produced more gesture-sign mismatches were more likely to succeed after instruction than those who did not. This adds to the evidence by suggesting that mismatches occur even within the same modality, and strengthens the claim that it is critical to observe children's gestures as a form of math-output regardless of the modality of their language. Additionally, this finding highlights that the proposed integrated model may be extended for populations who use manual languages as well, though future research is required to further support each proposed connection.

In addition to studying whether the knowledge children express in gesture can be made available to them, it is also important to understand whether an external observer is able to recognize the utility of children's gestures. In other words, how does the literature support the connection within the model between children's self-produced gestures and the math-input they receive? One such study investigated this connection by recruiting a set of teachers (*n* = 8) to work with 3rd and 4th graders (*n* =38) on mathematical equivalence problems (Goldin-Meadow and Singer, 2003). Specifically, each child completed a pre-test of six problems, and explained their solutions to an experimenter. The teacher watched this pretest to gain insight on the child's knowledge, but was given no information or instruction regarding gestures. Each teacher then provided instruction on a set of problems before the child completed another, comparable posttest. Results showed that teachers were more likely to have variation in their instructions (e.g., give additional strategies) to children who had used more gesture-speech mismatches during their initial explanations. Therefore, children's own gestures (math-output) inadvertently shaped their own learning environment by evoking further explanation and support from the teacher (math-input). Not only does this happen spontaneously, but research shows that when adults are instructed to watch children's gestures, it can amplify the amount of information they were able to glean from children's gestures (Kelly et al., 2002). Even when the instruction was subtle, included different domains of knowledge, or different aged children, these results held. Thus, it is both possible to pick up on the information children possess implicitly by watching their gestures, and respond to these gestures in ways that may specifically scaffold the children's knowledge. These findings strengthen the connection within the integrated model between children's own math-output informing new math-input.

In sum, prior research provides evidence that self-produced gestures may benefit children's own learning and problem solving. These studies support the proposed, integrated model in several specific ways. First, they emphasize the modeled connection between math-input in children's environment and the subsequent impacts the input has on their math performance and learning. Second, literature which uniquely considers spontaneous or instructed self-produced gestures allows for additional insight to be added to the model, such as the how individual differences in children's knowledge made lead to differences in children's use of gestures, or differences in the benefits of gesture use itself. The same results are not reported with similar methods which employ physical action, which suggests that these mechanisms are unique to gesture. Additionally, prior research underscores the importance of centralizing the child within the model, given that a learner's own math knowledge and cognitive abilities can change the utility of gesture. Lastly, there is evidence suggesting that children's gestures are an indicator of their knowledge, and that this form of math-output that can be used as a tool by adults. This crucial collection of studies provides the connection within our model between children's gestures as math-output impacting the mathematical input they receive from others. Taken together, these studies highlight the necessity of a model where children's self-produced gestures in math environments can be studied further.

## Gesture and Executive Function (EF)

Given the multi-faceted role of gesture in children's math environments, it is critical to examine how the current literature supports the model's proposed connections between gestures and children's EF. Research outside the domain of mathematics has linked gesture specifically to EF from an early age (e.g., gesture, language, and EF; Kuhn et al., 2014). As previously discussed, individual's gestures may show information about implicit knowledge that is not found in their speech (Broaders et al., 2007; Pine et al., 2007). By shifting this information outside the mind and onto the hands, gesture is commonly proposed as a mechanism by which the user can “lighten their cognitive load” (Goldin-Meadow et al., 2001; Wagner et al., 2004). The idea of cognitive load is often presented as an offloading of related memory resources. While previous work has not drawn explicit connections to components of EF, more recent work has begun to delineate how gesture may be related to each subcomponents of EF. Thus, in this section, we review the literature regarding gesture, and their implied or direct connections made to the subcomponents of EF presented within the integrated model.

### Working Memory

Working memory is a limited capacity sub-system of EF where information is temporarily held and processed during problem solving. On average, children use more gestures when faced with an explicit working memory demand (Delgado et al., 2011). The mechanistic connections between working memory and gesture are commonly discussed within the math and gesture literature.

For example, the aforementioned study by Goldin-Meadow et al. (2001) examined how children's memory could be impacted if they used gesture during some parts of the common math-equivalence task, but then were told to keep their hands still during other parts. Results indicated that participants performed better on the memory task when they were able to use gesture. This suggests the use of gesture allowed for a reduction of working memory load, compared when participants had to speak without gesturing. The authors suggest the use of gesture allowed for a reduction in working memory demands, allowing for a greater allocation of cognitive resources for the memory task, thereby improving performance. This same finding was found with adults. Using an updated, age-appropriate set of math problems to solve and explain as well as a harder set of memory items, adults were told they were allowed to use gesture only on some of their explanations. Similar to the children, the adults' performance was better when they were able to use gesture compared to when they only used speech, suggesting that both children and adults who use gesture while they speak would benefit in a reduction of working memory demands (Goldin-Meadow et al., 2001; Wagner et al., 2004). Thus, the current model reflects the direct connection between children's gestures and their working memory.

Ping and Goldin-Meadow (2010) further explored the mechanisms underlying how gestures benefit working memory. In this study, 2nd and 3rd grade children (M age 8.75 years) watched as an experimenter perform Piagetian conservation transformations. Children were asked to remember a list of words, then turned around to explain conservation to a new experimenter at another table. The new table was either empty or had the same conservation objects. This manipulation was critical as it allowed the researchers to test whether the cognitive benefits of gesture were based in its bodily capacity to link to a specific object or location (e.g., Ballard et al., 1997; Glenberg and Robertson, 1999). However, children who used gestures during their conservation explanations performed better on the memory task even when the items were absent and could not be directly indexed by gesture. Therefore, the working memory benefits of gesture are not tied to any specific object or spatial relation within the external environment.

More recent research with adults emphasizes the specific connection between gesture and working memory. For example, adults who are asked to use gesture may experience differential working memory benefits depending on their initial working memory abilities (Marstaller and Burianová, 2013). Additional studies have shown that people who have either lower visuospatial or verbal working memory capacity tend to produce more gestures on average (Chu et al., 2014; Gillespie et al., 2014; Pouw et al., 2016), and those who have higher than average visuospatial working memory abilities seem to be better equipped to detect information conveyed in gesture (Wu and Coulson, 2014a,b; Özer and Göksun, 2020). Thus, the connection between gesture and working memory are well-established.

The results of these studies are represented in the proposed model. Specifically, the proposed model reflects the bidirectional flow of information processing between children's own gestures and their working memory. This highlights the critical question of whether individual working memory abilities change how children receive gesture based math-input, as well as whether an individual's propensity to gesture could be impacted by their working memory abilities. In other words, would a child's initial working memory ability explain variability in their subsequent use of gesture within a math task?

Currently, there is not enough work available to answer this question. However, one recent study sought to address the related issue of whether the flow of information processing should vary based on a child's initial domain-general cognitive abilities. Specifically, recent research with preschoolers (*n* = 81) found that their spontaneous gestures and working memory were related to their performance on an age-appropriate math task (Gordon et al., 2021). However, children's gestures were not significantly related to their working memory after controlling for age. This work leaves room for future research to investigate this dynamic relation in further detail.

### Attention

Additional work has informed the connection in our model between gestures and attention, another sub-component of EF. Research with infants indicates that they attend to pointing gestures before 6-months of age (Rohlfing et al., 2012). Shortly after 1 year, they begin to make their own attention-directing gestures to convey and request information from other people in their environment (Tomasello et al., 2007; Kovács et al., 2014), suggesting at least a basic understanding of the attentional function of gesture. Therefore, within the proposed model, children could be expected to both use and recognize the utility of gesture as a tool for attention.

However, the primary function of gesture is not only to drive attention. For example, one of the previously described studies exposed children to math gestures that contained task-relevant information, but also that directed their attention to irrelevant components of the math problem (Goldin-Meadow et al., 2009). Results showed children who saw these partially-correct gestures still learn more than children who received no gestures at all, suggesting that even though their attention may have been drawn to less relevant components, the gestures still helped. Nevertheless, attention has still been added as its own separate component within the proposed model, given that children in this study still learned the most when they received a gesture that contained both the task-relevant strategy information and directed their attention to the relevant parts of the problem. Therefore, we still believed it is important to include within our model that gestural math-input can direct children's attention towards relevant information within their environment.

Recent research lends additional support to retaining attention in some way within the proposed model. Specifically, Wakefield et al. (2018) investigated how gestural input could change children's visual attention during math instruction. Eight- to ten-year-old children (*n* = 50) participated in the math equivalence paradigm and watched videos of a teacher's instruction in speech alone or in speech and gesture. Children's eye movements were captured using eye-tracking technology, and their learning progress as well as concept transfer was assessed. Children who received both speech and gesture instruction spent time looking at both the problem and the gestures. Additionally, children who received instructions with both speech and gesture were more likely to follow along visually^4^. Following along was uniquely predictive of learning for those in the speech and gesture condition. Therefore, gesture as math input appears to moderate the impact of visual attention on learning and provides additional support for the inclusion of a connection between gesture input and attention within the proposed model.

The current model also ties children's self-produced gestures to their attention. There are limited empirical examples that directly test how children's own gestures drive their attention in ways that impact their math output and learning. However, Alibali and Kita (2010) assessed whether prohibiting children's gestures would result in a shift of focus away from task-relevant information, which provides equal insight into this part of the model. In this study, researchers asked whether prohibiting 50 children (M age = 6 years, 5 months) from gesturing in the standard Piagetian conservation task would cause them to shift focus away from the perceptual-motor information which is commonly expressed in gesture. At first, all children were allowed to explain the conservation task with gesture, and then half the children were prohibited from gesturing for the second round of explanations by wearing a muff on their hands. On average, children were more likely to focus on information that was not perceptually present when they did not have access to gesture. When they were allowed to gesture, their focus shifted to the perceptually present information instead. Taken together, the results indicate that children's own gestures highlighted information within their own environment, and this information could be used in further cognitive processing related to children's later output. Therefore, while the main mechanism underlying gesture is not attention, it is still an essential component of EF that is tied to gesture. As such, the connection between gesture and attention within the proposed model are supported.

It is important to recognize that the proposed model does not include one connection built within the literature. Specifically, it has been suggested that individual speakers have a threshold for producing gestures, and that it may be possible for speakers to take advantage of this threshold (either directly or implicitly) to reap the cognitive benefits of gesture (Alibali and Nathan, 2012), suggesting a possible connection between attention sub-component of EF to gesture directly. The GSA framework provides a theoretical outlines how self-produced gestures are a consequence of a speaker's activation of own motor system involved in both planning and producing speech (Hostetter and Alibali, 2008). Based on a review of the empirical and theoretical supports, there is not enough support within the literature to draw a direct line from children's own math gestures to their own attention. As such, the proposed model only represents a flow of information routed by proxy of children's broader EF processes.

### Inhibitory Control

Although inhibitory control is an important component of EF, it is currently not included in our proposed model. This is, in part, because less is known about how gesture may impact or be impacted by a children's inhibitory control. Here, we briefly review two studies outside of the scope of the mathematics to highlight the potential for future research.

First, O'Neill and Miller (2013) examined preschool children's gestures (M age = 47 months) during a Dimensional Change Card Sort task. Children (*n* = 41) were asked first to sort cards based on a given rule (e.g., sort cards by color), then midway switched to sorting the cards by a new rule (e.g., sort by shape). To sort successfully, children must inhibit the first rule to sort by the new rule. In general, children who gestured more had higher performance. Similar to math tasks, children who used specific task-relevant gestures had higher performance compared to children who used less relevant gestures. In particular, the majority of differences were noted after the rule shift, which is when children would have needed to inhibit the old rule to implement the new rule.

Additional work with preschoolers using the same card sort task assessed whether a direct, causal relation existed between preschoolers' gestures and their scores on another version of the Dimensional Change Card Sort task (Rhoads et al., 2018). Specifically, preschoolers received training to use gesture as a support during the task to retain the specific dimensions they were using to sort. On average, children who were instructed to gesture showed improved sorting accuracy, and these instructions appeared to be particularly beneficial for younger children. These results suggest that instructing children to use gesture may boost their overall EF performance, or even lead to specific improvements in their inhibitory control abilities.

While these results occur outside of the domain of mathematics, they suggest that children's gestures may help to keep new rules in mind, inhibit an old rule, or some combination of the two. While the proposed model provides a breakdown of EF, and the information that flows between the sub-systems of attention shifting and working memory, further research needs to be conducted to better understand how to incorporate the third component of EF, inhibitory control, into the model.

## Discussion

In the current paper, we narrow our focus from the function of gesture across learning contexts broadly (e.g. Goldin-Meadow and Wagner, 2005) and present a new model regarding the role of gesture in math environments. The processes involved in math learning are well-modeled by the Information Processing Approach, however this approach is not able to fully explain the underlying mechanisms of gesture. Thus, we include tenets of Wilson's (2002) presentation of EC by modeling the mind within the body, and by extension the surrounding environment. This allows for a consideration of gestures as a form of math-input from the environment, as well as a form of math-output from children's own bodies. After the model presentation, we review the relevant literature on each of the model components. First, we briefly summarize the literature between math and EF, providing additional motivation for operationalization of IP into the sub-components of EF. Next, we review literature pertaining to gesture both as a form of math-input and math-output. Lastly, we summarize studies pertaining to the cognitive benefits of gestures, and how these relate to the sub-components of EF. Here, we outline the strengths and weaknesses of the proposed model and make recommendations for future research.

One strength of the proposed model is its direct expression of the connections that have been made separately, or alluded to, in previous literature. For example, the integrated model ties findings from studies of EF and math to those of gestures in a math context. In doing so, this new model presents a more holistic representation of the connections between gesture, and EF, and math. Specifically, given that EF and math abilities have been robustly linked throughout childhood (e.g., Bull and Espy, 2006), new studies should account for whether differences in EF's subcomponents change the contribution of gesture.

A related strength of the proposed model is its direct attribution of benefits of gesture directly to the specific, and separate components of EF (e.g., working memory, attention, inhibitory control) when necessary. The benefits of gesture are commonly described in terms of promoting conceptual change or providing cognitive supports. For example, self-produced gestures are often said to reduce cognitive burden, “thereby freeing up effort that can be allocated to other tasks” (Goldin-Meadow, 1999, p. 427). This reduction of “cognitive burden” or influence on is still broadly used to represent the complicated, tangle of cognitive processes that are relevant to discussing how, when, and why gestures are beneficial (e.g., Novack and Goldin-Meadow, 2017). While there is evidence of unidimensionality across EF constructs in infancy and early childhood (Wiebe et al., 2008; Hughes et al., 2010), much of the literature emphasizes the number of distinct and separate components of EF in later childhood and adulthood (see Baggetta and Alexander, 2016 for a review). Thus, this model is the first of its kind to outline the connections between gestures, math, and the potential of developing, multidimensional components of EF for children.

Another strength of the model is its capacity to represent how individual differences may impact the role gesture. A recent meta-analysis investigating the role of observed and produced gestures in comprehension found that while gestures are generally beneficial to comprehension, they are most beneficial when a learner produces gesture themselves (Dargue et al., 2019). Indeed, there are even times where gestures do not promote learning (see Goldin-Meadow, 2010 for a review). Therefore, the proposed model is unique in that it highlights how variation at the core of the model (e.g., the learner's EF abilities, math-knowledge stored in their long-term memory, and other factors which shape these capacities) will change when and for whom gestures will promote learning.

In addition to these strengths, there are several areas for future research that this model helps to identify. Specifically, the proposed model is primarily informed by gesture instruction and gesture use during two mathematical concepts, the mathematical-equivalence paradigm (Perry et al., 1988) and Piagetian conservation tasks (Church and Goldin-Meadow, 1986). To date, many math-gesture researchers have chosen to use these paradigms as they have been shown to produce natural and relevant gestures. Therefore, our model is heavily informed by studies which have repeatedly tested their questions within the same specific mathematical domain. As such, our model is limited in its scope in terms of representing gesture in a broader array of math contexts, ages, and levels of cognition. Future studies may examine how this model reflects gesture in mathematics more broadly. For example, while some studies have been conducted on early mathematical skills (counting and cardinality), more research on the benefits of gestures for foundational math knowledge is of particular importance given that children's early abilities are strongly linked to their later math achievement (Claesens and Engel, 2013; Geary et al., 2013; Watts et al., 2014). As such, it is imperative to understand how and when to use gesture in the mathematics classroom to best maximize academic achievement.

An additional consideration for the current model is how well it aligns with other proposed frameworks of gesture. While our model allows for gesture-speech mismatches produced or witnessed by children in a math environment, we do not center our model around them. However, we do not believe that the decentralization of gesture-speech mismatches in the proposed model conflicts with prior literature. For example, literature considering self-produced speech-gesture mismatches find that they are indicative of student's readiness to learn new information (Church and Goldin-Meadow, 1986). On the other hand, watching a teacher's mismatches may actually drive student's learning, compared to those who receive matching or no gestures (Singer and Goldin-Meadow, 2005). Thus, we argue it is not just that speech-gesture mismatches contribute to conceptual change that is important. Instead, our model prominently features the distinct pathways by which these mismatches could impact children's cognition, and how this impact may vary depending on the source.

One gap in the current model is its ability to address the neural underpinnings of gesture (e.g., Wakefield et al., 2013, 2019). This line of work is imperative and may provide additional insight regarding how each related brain region could play a role in learning. However, the proposed model does not provide a basis for studies considering detailed neurophysiological components. This is not to say that the results of these studies could not be thought of in parallel with the behavioral measures outlined within the proposed model. Rather, it is our goal to provide an accurate representation both in terms of the model's primary objective, as well as its scope. As such, while support for the proposed model may be further strengthened by neuroscience methods, it is possible that a more precise neural model of gesture use may be needed.

The implications and opportunities for future research within this domain are broad. Specifically, there are several questions remaining to be answered: How do the individual differences in children's EF impact their use gestures during math tasks? Additionally, how does children's level of math knowledge impact their EF, self-produced gesture, or the interaction between the two? Do the types and rates of gesture vary as a function of problem difficulty, based on these individual differences? How does the nature of these relations change as children's math knowledge grows, and the specific content they are learning changes? Although each of these questions are motivated from the substantial research on children's gesture, mathematics, and executive function, key information is still missing.

As discussed previously, one gap in the literature is how children's inhibitory control may be linked to the mechanisms and benefits of gestures. Math contexts are particularly useful to study how children employ their inhibition abilities. For example, during problems solving children can inhibit old, ineffective, or incorrect strategies in lieu of new or correct strategies they have learned more recently (Siegler, 1996). Thus, future research could analyze how gesture may be used to support strategy inhibition during these critical learning periods. Additionally, children's spontaneous gestures could provide insight into their inhibition. If a child produces old, ineffective strategy knowledge in speech but newer strategy knowledge in gesture, this mismatch could imply that supporting their inhibitory control abilities would allow them to use the strategy knowledge they displayed in their gestures.

Additional research could be conducted to better support the proposed model's connection between the math knowledge stored within children's long-term memory and their self-produced gestures. The proposed model follows the current literature in that math knowledge can be displayed in children's self-produced gestures (e.g., Garber et al., 1998), and an assessment of this “implicit” knowledge can be used to determine whether children are ready to learn (Broaders et al., 2007), thereby leading to additional math-input. Thus, a unidirectional arrow leads from the information stored in children's long-term memory to their gestures, but these gestures loop out into the environment to inform their math-input. Thus, future research could directly investigate how this information changes depending on if children's gestures were spontaneous or the result of instruction. For example, while these types of gestures may appear to display similar information, it is possible that the underlying reason why gestures are generated in these circumstances could vary. Additionally, a child's propensity to gesture could differ based on the instructions they receive, and therefore the types and rates of self-produced gestures could also be expected to differ.

Relatedly, future research could examine the differences between when children receive specific instruction to use gestures themselves, compared to when they are just broadly exposed to gesture and mimic these movements independently. In other words, if a child is exposed to a particular type of gesture in a math context, what could we expect from them in later math settings? Would the presenter of that gesture matter in terms of whether it was a parent, teacher, or even a peer? In the event that children are told about the specific benefits of using gesture as a tool for math, would children use it in a way that helps them? Or would they over-employ gesture in ways that hinders performance? These are just a few of the many questions that the proposed model is uniquely suited to address. In particular, it allows future researchers to question how gesture-based math-input may facilitate learning, while simultaneously considering children's EF, math knowledge, and their own gestures and math-output.

## Conclusion

The proposed model fuses central components of embodied cognition and information processing theories to highlight connections drawn in previous studies investigating gestures, EF, and math learning. Each component of this new model is outlined in a thorough review of the prior literature, through a combined lens of these two theories. Although there are several existing models of gestures and math learning, our model offers specific, novel avenues for future research. In particular, it provides a cohesive, theory driven representation of the role of gestures as they pertain to children's cognition within a math environment. In sum the proposed model provides future researchers with a theoretical foundation from which they may continue to understand the relations between gestures, EF, and children's math learning.

## Author Contributions

RG and GR developed the conceptual ideas. RG performed the literature search and drafted the manuscript. GR revised the manuscript and provided critical feedback for important intellectual content. All authors approved the submitted version.

## Conflict of Interest

The authors declare that the research was conducted in the absence of any commercial or financial relationships that could be construed as a potential conflict of interest.

## Footnotes

^1^An example of a problem involving the concept of math equivalence: in the problem 4 + 5 + 6 = _ + 6, children must recognize that the equals sign represents that one side should be equal to the other side, and that the problem requires them to solve for the blank within the equation.

^2^Children in the no-gesture group only had the opportunity to verbalize the relevant equivalence strategy. Children in the correct gesture group learned to use their fingers to group the two, specific numbers that should be added to get to the correct answer. Children in the partially correct gesture condition still used a grouping gesture but with two numbers that would not sum to the correct answer.

^3^In the original work, when children's speech and gesture contained different information, it was labeled as “discordant”, and if they contained the same information it was termed “concordant”. However, these terms are used less commonly today, and we have replaced them to be consistent in our terminology across reference of this concept.

^4^For example, the instructor says “one side equal to the other side” while pointing the specific sides of the problem referenced in speech, and the child switches their gaze to the indicated components of the problem.
